# Resin Composite Surface Pre-Reacted Glass-Ionomer (S-PRG) Filler for Non-Carious Cervical Lesions: A Double-Blinded, Randomized, Split-Mouth Clinical Trial

**DOI:** 10.3390/dj13040156

**Published:** 2025-04-01

**Authors:** Adam Lowenstein, Carlos Fernando Mourão, Mabi L. Singh, Sarah E. Pagni, Ronald D. Perry, Gerard Kugel

**Affiliations:** 1 Department of Basic and Clinical Translational Sciences, Tufts University School of Dental Medicine, Boston, MA 02111, USA; carlos.mourao@tufts.edu; 2 Division of Oral Medicine, Department of Diagnostic Sciences, Tufts University School of Dental Medicine, 1 Kneeland St, Boston, MA 02111, USA; mabi_l.singh@tufts.edu; 3 Department of Public Health and Community Service, Tufts University School of Dental Medicine, 1 Kneeland St, Boston, MA 02111, USA; sarah.pagni@tufts.edu; 4 Department of Comprehensive Care, Tufts University School of Dental Medicine, 1 Kneeland St, Boston, MA 02111, USA; ronald.perry@tufts.edu; 5 College of Dentistry, University of Nebraska Medical Center, Omaha, NE 68198, USA; gkugel@unmc.edu

**Keywords:** restorative material, dental materials, operative dentistry, composite

## Abstract

**Background/Objectives**: This double-blinded study aimed to evaluate the clinical performance of a giomer restorative material in comparison to a nanotechnology-based restorative system for the restoration of non-carious cervical lesions over a period of 48 months. **Methods**: A 48-month randomized, controlled, split-mouth trial was conducted with 49 subjects at its baseline, which was reduced to 34 subjects at follow-up, yielding a statistical power of 69.55%. Cervical lesions were restored using either BEAUTIFIL II LS (BL) or 3M/ESPE Filtek Supreme Universal Restorative (FS). Clinical assessments were performed by blinded examiners, excluding the one who placed the restorations. Evaluations were based on the Hickel criteria, covering esthetic, functional, and biological properties, with comparisons made using the Wilcoxon signed-rank test. **Results**: Hickel scores were analyzed with the Mann–Whitney U test to compare BL and FS groups within subjects. Changes in median Hickel scores, sensitivity, and the gingival index were assessed via Friedman’s test, followed by Wilcoxon signed-rank tests with a Bonferroni correction for post-hoc comparisons. The p-values under 0.05 were considered significant, except with the Bonferroni correction. Statistical analysis showed no significant differences between materials across most Hickel parameters (*p* > 0.05), though BL exhibited a trend of better surface staining (76.5% excellent with BL vs. 76.5% excellent with FS) and adjacent mucosa, while FS showed slight advantages in surface luster and color match. **Conclusions**: The clinical evaluation of restorations for non-carious cervical lesions using giomer and nanotechnology-based restorative systems revealed no statistically significant differences according to the Hickel criteria, indicating a similar clinical performance for both restorative materials.

## 1. Introduction

Non-carious cervical lesions (NCCLs), characterized by the loss of tooth structure at the cemento-enamel junction (CEJ), are frequently encountered in clinical dental practice. These lesions have non-bacterial origins and are particularly prevalent among the aging population who have retained their natural teeth for an extended period. The primary etiology of NCCLs is mechanical abrasion, often attributed to the abfraction of hard tissue at the CEJ [1,2]. Additionally, occlusal stresses concentrated in the cervical region have been proposed as contributing factors to the failure of restorations in this area, leading to issues such as debonding, leakage, and, ultimately, restorative failure [3].

Clinically, NCCLs can manifest as shallow or deep depressions and disc- or wedge-shaped defects at the CEJ [2]. The implications of these lesions are significant, as they result in the loss of tooth structure, which can weaken the overall dentition. Furthermore, these structural defects can accumulate plaque and serve as reservoirs for bacterial colonization [1]. Depending on the extent of structural loss, patients may experience hypersensitivity, and the vitality of the pulp can be compromised due to the bacterial sources present in plaque [4]. Moreover, cervical lesions that manifest defects at the CEJ can adversely affect esthetics, underscoring the importance of restoring normal structural anatomy in Class V restorations [5].

Methacrylate-based composites have emerged as the gold standard for direct restorative procedures, owing to their superior esthetic and mechanical properties compared to glass-ionomer cements or hybrid-ionomers [6]. Recent advancements in understanding the etiology of NCCLs and Class V restorations have led to clinical approaches that integrate chemical adhesion with restorative materials possessing appropriate mechanical properties, particularly in terms of fracture toughness and elasticity. These approaches show promise for achieving long-term success in restorative outcomes [2,6].

In clinical practice, the preparation of cavities for NCCLs presents a challenge to creating a retention shape due to the limited amount of healthy tooth structure available between the cervical lesion surface and the pulp. Consequently, cavity preparations often focus on the removal of sclerotic dentin to optimize the bonding surface while minimizing the overall loss of tooth structure [7]. Unlike traditional G.V. Black cavity preparations, Class V resin composite restorations primarily depend on the bonding strength between the restorative material and the enamel/dentin interface for retention. Therefore, the selection of optimal materials is critical for achieving the best clinical outcomes, which include restoring esthetics, preventing further tooth structure loss due to abfractions and erosion, and insulating and protecting the pulp [8].

One of the materials evaluated is Beautifil II LS (BL), a nanohybrid restorative material developed by Shofu (Kyoto, Japan), which features a polymer matrix composed of low-shrinkage urethane diacrylate, Bisphenol A polyethoxy methacrylate (Bis-MPEPP), Bisphenol A diglycidylmethacrylate (Bis-GMA), and triethylene glycol dimethacrylate (TEGDMA) [7]. A notable characteristic of this restorative material is its bioactive surface pre-reacted glass-ionomer (S-PRG) filler, which, in conjunction with the polymer matrix, is reported to exhibit low volumetric shrinkage and shrinkage stress [3].

The bonding system associated with Beautifil II LS, known as BeautiBond, is an acetone/water solvent-based system that incorporates phosphonic acid and carboxylic acid monomers, which are optimized for bonding to enamel and dentin, respectively [7]. In contrast, the second restorative material under investigation is the 3M/ESPE Filtek Supreme Universal Restorative (FS), paired with the Scotchbond™ Universal Adhesive by 3M (St. Paul, MN). This material is formulated from a combination of Bis-GMA, urethane dimethacrylate (UDMA), TEGDMA, and Bisphenol A polyethylene glycol diether dimethacrylate (Bis-EMA-6), with poly(ethylene glycol) dimethacrylate (PEGDMA) included to reduce the shrinkage rate [9].

The S-PRG filler in BL releases fluoride, strontium, sodium, borate, aluminum, and silicate ions, which laboratory studies suggest could enhance clinical outcomes in NCCLs. Remineralization is a key potential benefit, as fluoride and strontium may promote mineral deposition on dentin surfaces, strengthening tooth structure and reducing hypersensitivity—a common issue in NCCLs. The antimicrobial effects, driven by ion release, could inhibit bacterial colonization at restoration margins, where plaque accumulation is a concern due to the cervical location. Acid neutralization by these ions may counteract the acidic environment from dietary or bacterial sources, potentially reducing the secondary caries risk [5,6,7]. The comparative analysis of these two restorative material systems in a clinical trial setting is expected to yield valuable insights for clinicians in selecting the most effective materials for optimal clinical outcomes. The Beautifil II LS system is anticipated to provide unique advantages due to its bioactive filler, which may enhance remineralization and exhibit antipathogenic effects while also demonstrating a low shrinkage rate. Conversely, the nano-sized filler-based Filtek Supreme system is expected to excel in esthetic properties, material strength, and bonding performance [2,6].

Previous clinical trials, such as Gordan et al., reported a similar performance between giomer and conventional composites over 13 years, while Kubo et al. found 83–100% survival rates for hybrid resins in NCCLs over 3 years [10,11]. Short-term studies of S-PRG fillers showed high success, yet long-term comparisons with nano-filled composites like FS remain scarce, justifying this study [12].

The objective of this clinical trial was to evaluate the effectiveness of these composite resin systems in restoring NCCL Class V lesions. A 48-month split-mouth clinical trial design was employed, with restorations assessed according to the survival rate guidelines. Comprehensive clinical performance was evaluated using the Modified Hickel Evaluation Criteria. It was hypothesized that both restoration systems would perform comparably under the Hickel grading system, leading to the establishment of the null hypothesis: H_0_: BL and FS Hickel gradings are equal. These findings guided clinicians in selecting materials for NCCLs, balancing esthetic demands with potential bioactive benefits in caries-prone or mechanically stressed cases.

## 2. Materials and Methods

### 2.1. Ethical Considerations

**Figure 1:**
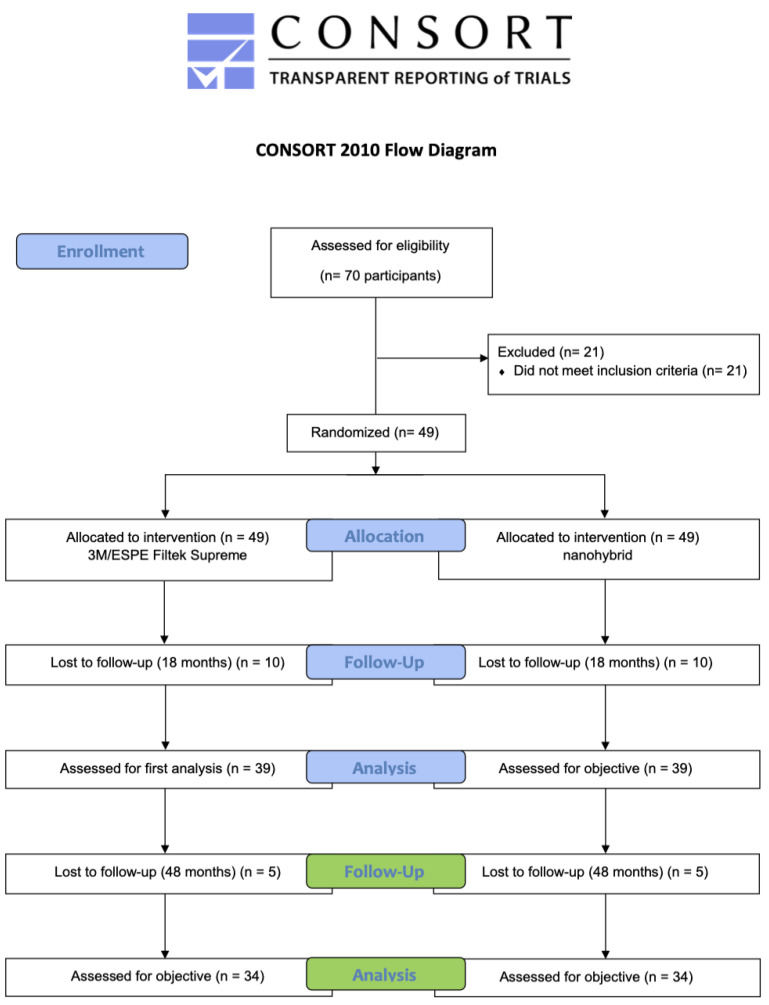
CONSORT flow chart diagram.

This study was designed as a randomized, controlled, split-mouth trial conducted in accordance with the principles outlined in the Declaration of Helsinki regarding experiments involving human subjects and in compliance with Normative Resolution No. 466 of 2012 from the National Health Council. The research protocol received approval from the Tufts University School of Dental Medicine Institutional Review Board (IRB #12486, approval date: 5 May 2017). Additionally, the clinical trial was registered with ClinicalTrials.gov (NCT03153969, registration date 17 May 2017). The study adhered to the CONSORT statement guidelines to ensure quality and transparency throughout the randomized trial process. Subject volunteers were recruited after being provided informed consent by signing the Informed Consent Form (ICF), which outlined the study’s guidelines and schedule (Figure 1). The clinical study occurred at the Tufts University School of Dental Medicine (TUSDM) Research Clinic in Boston, MA. This 48-month randomized, controlled, split-mouth clinical trial was structured over four visits: screening and restoration placement (0-month) and follow-ups at 6-month, 18-month, and 48-month intervals. The current study examined and analyzed data collected at 48 months. At the request of the consenting subjects, the screening and restoration visits were occasionally combined into a single appointment. All examiners were blinded and underwent calibration and training before the commencement of the study. Patients were blinded to the restorative material (BL or FS) assigned to each lesion, alongside evaluating clinicians, to minimize bias in subjective outcomes, like patient satisfaction.

### 2.2. Sample Size Calculation and Randomization

A sample size of 49 subjects was used based on a previous study [12] that demonstrated results after 18 months and determined that—based on the assumption that modified Hickel scores for the control composite would show that 50% of restorations would receive a score of 1, 40% a score of 2, and 10% a score of 3, with no restorations scoring 4 or 5 at the 18-month mark—this sample size was sufficient to achieve 80% power [12]. In contrast, the modified Hickel scores for the resin composite were anticipated to indicate that 77.8% of restorations would score 1, 22.2% would score 2, and none would score 3, 4, or 5 at 18 months. The calculations accounted for a type I error rate of 5% and a dropout rate of 20%. The randomization of subjects was performed using an Excel file (Microsoft Excel Version 16.95.1, Mountain View, CA, USA).

### 2.3. Inclusion and Exclusion Criteria

The inclusion criteria for subjects in this study required participants to have a minimum of two cervical lesions necessitating restoration. Each cervical lesion had to allow for at least 1 mm of restorative material while preserving the natural contour of the tooth, with at least 50% of the lesion involving dentin and the coronal margin remaining in the enamel.

Exclusion criteria included the presence of periapical pathology or symptoms indicative of pulpal pathology. Additionally, teeth that were non-vital or had undergone root canal therapy; those exhibiting near pulp exposure, as determined by radiographs; that were hypersensitive; had advanced periodontal disease; and had abutments for removable prostheses were excluded from participation.

### 2.4. Recruitment of Subjects and Clinical Procedures

Two clinicians performed the placement of 98 Class V restorations across 49 subjects. Each subject received two Class V restorations; one NCCL was randomly assigned to be restored with BL, while the other was restored with FS, utilizing the total-etch method for both restorations. The compositions of the materials used are detailed in Table 1. The age range of subjects was between 27 and 80 years. All participants were provided with a written ICF and consented by signing to participate in the study.

**Table 1:** Material composition and comparisons (table adapted from Kang et al. 2021 [12]).

Shofu BL II LS [11]	3M FS Supreme Universal [13]
Low-shrinkage urethane diacrylate, Bis-GMA, Bis-MPEPP, TEGDMA, S-PRG filler, multi-functional glass filler, pre-polymerized filler, nano-filler, photoinitiator, etc.	Bis-GMA, UDMA, TEGDMA, Bis-EMA(6) non-agglomerated/non-aggregated 20 nm silica filler, non-agglomerated/non-aggregated zirconia filler, and aggregated zirconia/silica cluster filler
**BeautiBond [14]**	**3M Scotch Universal Bond [15]**
Acetone, Bis-GMA, TEGDMA, phosphonic acid monomer, carboxylic acid monomer, water, photoinitiator, polymeric monomer, HEMA-free, etc.	MDP phosphate monomer, dimethacrylate resins, HEMA, Vitrebond™ copolymer, filler, ethanol, water, initiators, silane

For this study, NCCLs were prepared conservatively to preserve as much healthy dentin and enamel as possible. Diamond burs (Brasseler USA, Savannah, GA, USA) were initially employed, followed by finishing with non-fluoride pumice to prepare the cavities. Isolation was achieved using Isolite (Zyris, Santa Barbara, CA, USA) or Mr. Thirsty (Zirc, Buffalo, MN, USA) in conjunction with cotton rolls. The bonding procedure utilized the total-etch method, after which the assigned composite materials were placed to restore the prepared lesions. Completed restorations were finished and polished using the Shofu Super Snap Rainbow Technique Kit (Shofu, Kyoto, Japan). Clinical images were captured before, after, and during follow-up visits using the EyeSpecial C-II (Shofu, Kyoto, Japan).

Clinical assessments using the modified Hickel criteria were completed [16]. Efforts were made to ensure that the same examiner evaluated the same subject at each visit. Restorations were assessed based on modified clinical criteria established by Hickel et al., which included esthetic properties (surface luster, surface staining, marginal staining, color match, and anatomical form), functional properties (material fracture and retention, marginal adaptation, and subject’s view), and biological properties (recurrence of caries, tooth integrity, and adjacent mucosa). Evaluation grading criteria are provided in Table S1.

Descriptive statistics were calculated, and at each time point, differences in median Hickel scores, sensitivity, and the gingival index were compared between products using the Mann–Whitney U test. For each product, changes in median Hickel scores, sensitivity, and gingival index were assessed using Friedman’s test, followed by the Wilcoxon signed-rank test with a Bonferroni correction for post-hoc pairwise comparisons. A *p*-value less than 0.05 was considered statistically significant, except when the Bonferroni correction was applied. Statistical analyses were performed using Stata 17 and SPSS 28.

## 3. Results

This split-mouth study started with 49 subjects, each with two restorations placed under the established TUSDM standard of care, totaling 98 restorations at the baseline visits. At the 48-month recall examination, 34 patients, totalizing 68 (69.4%) restorations, were evaluated, and socio-demographic data were recorded (Table 2).

At 48 months, the power calculation differed due to the drop-off of subjects. The final sample size was 34 subjects. Using that data and the final sample size, a power of 69.55% was observed for the 48-month time point.

**Figure 2:**
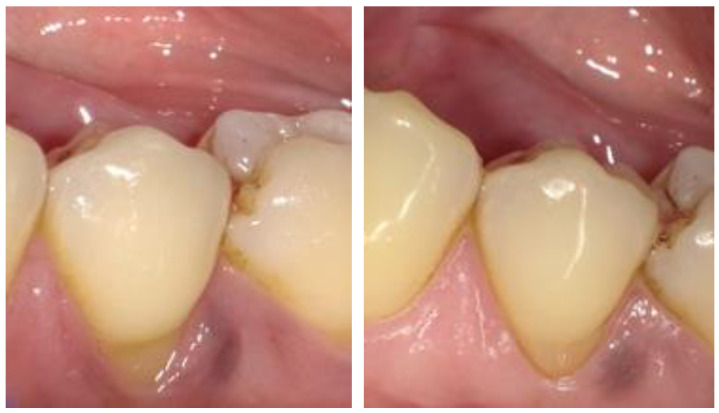
Clinical image of BL at baseline (**left**) and 48 months (**right**).

**Figure 3:**
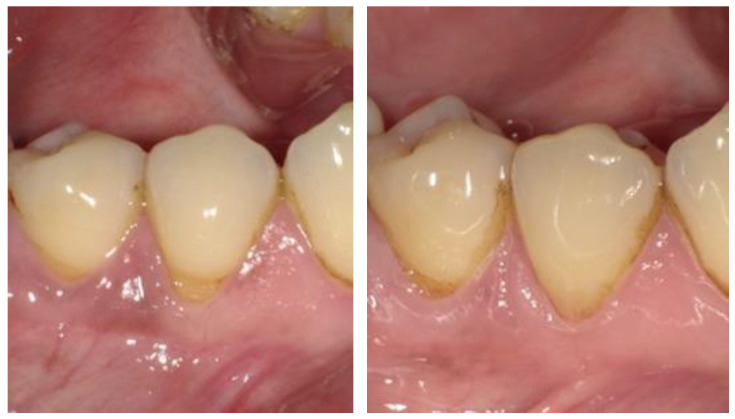
Clinical image of FS at baseline (**left**) and 48 months (**right**).

Clinical evaluations conducted using the Hickel criteria (Table 3) indicated comparable performance between the two material systems, with scores ranging from 1, denoting clinically excellent/very good, to 5, indicating clinically poor (replacement necessary). The distribution of Hickel scores and the material performance comparison at baseline and at 48 months are detailed in Table 4 and Table 5. Both restoration systems demonstrated similar outcomes when examining the number of cases that received a clinically excellent/very good rating. A 100% occurrence rate for a score of 1 (clinically excellent/very good) was observed in the categories of postoperative sensitivity, recurrence of caries, erosion and abfraction, and tooth integrity. Additionally, both restorative systems performed comparably across all other categories. However, it is noteworthy that the categories of marginal staining and marginal adaptation exhibited occurrence rates at or below 80%, suggesting that the marginal seal may deteriorate under cyclic flexural deformation of the tooth, adversely affecting the bonding between the restorative material and the tooth structure. Representative clinical photographs for both BL and FS taken at each visit are displayed in Figure 2 and Figure 3.

Degradation in marginal staining (BL: 100% to 64.7%, FS: 98% to 64.7%) and adaptation (BL: 98% to 55.9%, FS: 100% to 52.9%) showed no significant between-material differences (*p* > 0.05) despite similar temporal declines (*p* < 0.001). In Table 4, asterisks marked statistical significance.

## 4. Discussion

This study aimed to evaluate the long-term clinical performance of two restorative materials, BL and FS, in the context of NCCLs classified as Class V restorations. Prior to enrollment, participants underwent a thorough screening process to confirm their general health and the absence of medical conditions that could potentially affect the restorative materials’ performance. This included a comprehensive oral examination assessing various periodontal health indicators, such as gingival inflammation, recession, clinical attachment levels, probing pocket depth, and calculus presence, ensuring that participants were in optimal periodontal health [17]. This rigorous assessment is crucial, as periodontal health can significantly influence the longevity and success of dental restorations [18].

Our previous study—the 18-month follow-up—revealed high success rates for both restorative systems, with BL and FS showing retention rates of 90.7% and 95.3%, respectively [12]. However, the 48-month follow-up indicated a slight decline in these rates, with BL restorations decreasing to 88.2% and FS to 94.1%. This trend suggests that, while both materials demonstrate durability over time, they are not immune to gradual degradation, likely due to cyclic flexural stresses that compromise marginal integrity [10]. The reduced sample size at the 48-month mark, attributed to participant drop-off, resulted in a statistical power of 69.55%, which may have impacted the robustness of our findings. Nevertheless, clinical evaluations using the Hickel Criteria showed comparable long-term performance between the two materials, with both maintaining high ratings across critical categories, such as postoperative sensitivity, caries recurrence, erosion, and tooth integrity [19].

Regarding the Hickel scores, the lower occurrence rates of excellent ratings for marginal staining (64.7%) and adaptation (55.9% BL/52.9% FS) at 48 months suggest gradual deterioration that may impact long-term restoration success. Marginal integrity and staining are critical predictors of future failure, particularly when combined, yet their clinical implications in this study remain subtle, given the absence of significant secondary caries or retention loss. Patient-specific factors, such as poor oral hygiene or high occlusal stress, likely contributed to these outcomes [16].

NCCLs pose a significant challenge in restorative dentistry, particularly as their prevalence increases among an aging population that retains natural dentition longer [1]. The unique biomechanical environment of NCCLs, where occlusal stresses are concentrated at the cervical region, complicates restoration efforts, often leading to failure through debonding and leakage [10]. The introduction of surface pre-reacted glass-ionomer (S-PRG) filler technology offers a promising alternative due to its ability to release multiple ions, including strontium, borate, fluoride, sodium, silicate, and aluminum, which can enhance the restorative material’s performance [19]. This bioactive material not only exhibits lower polymerization shrinkage compared to conventional composites but also provides potential benefits in terms of tooth strengthening, acid neutralization, and antimicrobial properties [10]. Despite the encouraging laboratory results for S-PRG filler-containing materials, there remains a pressing need for long-term clinical evidence to substantiate their efficacy against established nanotechnology-based composites in the challenging context of cervical lesions [12].

The retention rates observed in this study were consistent with previous literature [12], with BL and FS achieving retention rates of 88.2% and 94.1% at the 48-month follow-up, respectively. While the American Dental Association guidelines indicate that a retention rate of 90% at 18 months signifies full acceptance, they do not provide benchmarks for longer durations [1]. The retention levels reported here align with findings from other studies, which have documented varying survival rates for Class V restorations, ranging from 83% to 100% [19]. The variability in retention rates can be attributed to differences in preparation techniques and the inherent challenges associated with NCCLs, which lack sufficient retention form, thus relying heavily on the bond strength of the restorative materials to enamel and dentin [19]. The integrity of the marginal seal is pivotal in preserving this bond, as evidenced by the Hickel criteria evaluations indicating a similar performance between both materials in terms of marginal staining and adaptation [17].

Compared to Gordan et al.’s 13-year giomer trial (with similar performance to composites) and Kubo et al.’s 3-year study (83–100% survival), our 48-month findings align with stable long-term outcomes, though our study showed marginal degradation that exceeded shorter-term reports. Nano-filled composites like FS consistently excel in esthetics, while giomers’ bioactive benefits remain theoretical without longer follow-ups [10,11,19].

The decline in marginal staining and adaptation (below 80% excellent ratings) suggests its vulnerability to occlusal stress and biochemical degradation. Improvements could include using adhesive systems with enhanced bond strength (e.g., self-etch primers with higher dentin affinity), incorporating low-shrinkage resins to reduce interfacial stress, and advising patients on occlusal management (e.g., nightguards for bruxism) and oral hygiene to minimize staining. For BL, leveraging its S-PRG ion release to prevent demineralization at margins could be optimized with longer observation periods or adjunctive fluoride therapies.

Comparative analysis of the Hickel scoring between the 18-month and 48-month follow-ups revealed minor changes in clinical performance for both BL and FS restorations. At the 48-month recall, the distribution of scores indicated a slight decline in the percentage of restorations rated as clinically excellent or very good, reflecting the gradual degradation of restorative materials over time [9]. The Wilcoxon signed-rank test confirmed that these changes were not statistically significant, suggesting that both materials maintained comparable clinical performance despite the observed trends [12].

The early deterioration of marginal seals in Class V restorations may be attributed to a combination of biochemical factors related to caries-inducing bacteria and mechanical stresses from occlusion and bruxism. Enhancing marginal adaptation and preventing secondary caries at the margins are critical for improving the overall retention rate of Class V restorations. The ion-releasing capabilities of S-PRG fillers in BL materials may play a crucial role in mitigating secondary caries by continuously releasing ions that help prevent demineralization at the restoration margins.

A limitation of light-curing composite resin materials is their susceptibility to polymerization shrinkage, particularly at the tooth–composite interface. The reported volumetric shrinkage for conventional composites ranges from 1.9% to 13.5%, whereas BL exhibits a significantly lower shrinkage of 0.85%. This reduced shrinkage results in less residual stress at the interface, thereby enhancing interfacial bonding to enamel and dentin. Furthermore, the potential for bioactive bonding formation in BL, facilitated by the release of key ions from S-PRG fillers, has been shown to promote mineral formation on the dentin surface, further enhancing the material’s clinical performance.

The dropout rate of 30.6% (from 49 to 34 subjects) reduced statistical power, potentially limiting the study’s ability to detect small but clinically relevant differences between BL and FS. Future studies could mitigate this by recruiting a larger initial sample size to account for attrition, implementing retention strategies, or using statistical adjustments to better handle missing data and maintain power over long-term follow-ups. The lack of sensitivity analyses limit adjustments for dropout; such methods could assess the robustness of findings despite reduced power.

Esthetic considerations, such as color match and translucency, are critical in the clinical evaluation of Class V restorations, particularly since these lesions are often located in highly visible areas. The subjective nature of esthetic evaluations, as evidenced by the variability in Hickel scores among evaluators, underscores the challenges in achieving consistent assessments of color match and translucency. Despite these challenges, nanocomposites like FS have demonstrated superior esthetic properties, including improved resistance to micro-abrasion, which may help prevent staining as the restoration ages.

While both restorative materials demonstrated comparable clinical performance over the 48-month period, the nuances in their properties and performance highlight the importance of ongoing evaluation and monitoring. Future studies should explore the performance of these materials in more challenging clinical conditions, particularly among populations at higher risk for caries, to better understand their long-term efficacy and potential advantages in restorative dentistry.

Our findings indicate comparable performance between BL and FS over 48 months, with subtle differences applicable to clinical practice. FS may be preferred for anterior NCCLs requiring superior esthetics given its slight advantages in surface luster (88.2% excellent for FS vs. 79.4% excellent for BL) and color match (67.6% for FS vs. 70.6% for BL). BL could be considered for patients with subgingival lesions or a higher caries risk, where the S-PRG filler’s theoretical remineralization and antimicrobial effects might offer benefits, though these were not statistically evident here. Clinicians should weigh these factors alongside cost, handling preferences, and patient-specific needs (e.g., occlusal load), as differences appear clinically minor within this timeframe.

## 5. Conclusions

The findings from this clinical study indicated that the nanohybrid composite containing S-PRG filler, Shofu Beautifil II LS, exhibited clinical performance comparable to that of the nanocomposite 3M Filtek Supreme, as assessed by the modified Hickel criteria. The study’s statistical power was reduced to 69.55% due to a 30.6% participant dropout rate, which may limit its ability to detect smaller differences between groups. Older adults, who have often retained teeth longer and face increased NCCL prevalence, might benefit from either material. These distinctions lack strong evidence, warranting further research into patient-specific outcomes. No statistically significant differences were observed across any of the evaluated categories after 48 months.

## Figures and Tables

**Table 2:** Socio-demographic data of the patients included in the study at the 48-month follow-up.

Participants	*n* = 34
Sex	
Male	18
Female	16
Age (years), Mean ± Standard Deviation	55.88 ± 14.72
Smoker	5
Ethnicity	
Native American	0
Hispanic/Latino	3
Asian	5
Black	7
White	16
Prefer Not to Answer	3

**Table 3:** Hickel scoring comparison at baseline and 48 months.

	Hickel Criteria	Visit	BL Hickel Scores					FS Hickel Scores				
			1	2	3	4	5	1	2	3	4	5
Esthetic Properties	Surface Luster	Baseline	49					49				
		48 months	30	3				27	4	1		
	Surface Staining	Baseline	49					49				
		48 months	26	6	1			26	5	1		
	Marginal Staining	Baseline	49					48	1			
		48 months	22	7	3	1		22	7	1	2	
	Color Match	Baseline	46	3				44	5			
		48 months	24	6	2	1		23	6	2	1	
	Anatomical Form	Baseline	48	1				49				
		48 months	33					30	1	1		
Functional Properties	Fracture of Material and Retention	Baseline	49					49				
		48 months	33				1	31			1	1
	Marginal Adaptation	Baseline	48	1				49				
		48 months	19	13		1		18	12	1	1	
	Patient’s View	Baseline	49					49				
		48 months	32	1				31	1			
Biological Properties	Recurrence of Caries	Baseline	49					49				
		48 months	27	1	1	3	1	26	4	1	1	
	Tooth Integrity	Baseline	49					49				
		48 months	32		1			32				
	Adjacent Mucosa	Baseline	48	1				48	1			
		48 months	21	8	5			23	6	4		

**Table 4:** Hickel scoring comparison at baseline and 48 months. * statistical significance.

		BL Hickel Scores			FS Hickel Scores		
	Hickel Criteria	Baseline Median (IQR)	48 Months Median (IQR)	*p*-Value	Baseline Median (IQR)	48 Months Median (IQR)	*p*-Value
Esthetic	Surface Luster	1 (0)	1 (0)	0.11	1 (0)	1 (0)	0.07
	Surface Staining	1 (0)	1 (0)	0.8	1 (0)	1 (0)	0.03 *
	Marginal Staining	1 (0)	1 (1)	<0.001 *	1 (0)	1 (1)	0.008 *
	Color Match	1 (0)	1 (0)	0.12	1 (0)	1 (0.5)	0.09
	Anatomical Form	1 (0)	1 (0)	>0.99	1 (0)	1 (0)	>0.99
Functional	Fracture of Material and Retention	1 (0)	1 (0)	>0.99	1 (0)	1 (0)	>0.99
	Marginal Adaptation	1 (0)	1 (1)	<0.001 *	1 (0)	1 (1)	<0.001 *
	Patient’s View	1 (0)	1 (1)	>0.99	1 (0)	1 (1)	>0.99
Biological	Recurrence of Caries	1 (0)	1 (0)	0.01 *	1 (0)	1 (0)	0.04 *
	Tooth Integrity	1 (0)	1 (0)	>0.99	1 (0)	1 (0)	>0.99
	Adjacent Mucosa	1 (0)	1 (1)	<0.001 *	1 (0)	1 (1)	<0.001 *

**Table 5:** Material performance comparison.

	0 Month	48 Months
Surface Luster	=	FS
Surface Stain	=	BL
Marginal Stain	FS	FS
Color Match and Translucency	FS	FS
Esthetic Anatomical Form	BL	FS
Fracture of Material and Retention	=	FS
Marginal Adaptation	BL	FS
Radiographic Examination (when applicable)	n/a	=
Patient’s View	=	=
Postoperative (Hyper-)Sensitivity and Tooth Vitality	n/a	=
Recurrence of Caries, Erosion, Abfraction	=	=
Tooth Integrity (Enamel Cracks, Tooth Fractures)	=	=
Adjacent Mucosa	=	BL

## Data Availability

Data supporting the findings of this study are not publicly available due to privacy concerns. Further details about the data and conditions for access can be obtained from the corresponding author upon reasonable request.

## Supplementary Materials

The following supporting information can be downloaded at: www.mdpi.com/article/10.3390/dj13040156/s1, Table S1. Scoring Criteria for Direct Assessment from Modified Hickel Criteria (Table adapted from Kang et al. [12]).
