# Supplementary material for: Resin Composite Surface Pre-Reacted Glass-Ionomer (S-PRG) Filler for Non-Carious Cervical Lesions: A Double-Blinded, Randomized, Split-Mouth Clinical Trial
Source: Dent J (Basel). 2025 Apr 1;13(4):156. doi: 10.3390/dj13040156 (PMC12025950; doi:10.3390/dj13040156)
Supplement: Supplementary file 1 [file dentistry-13-00156-s001.zip › dentistry-3447076-supplementary.pdf]

Table S1. Scoring Criteria for Direct Assessment from Modified Hickel Criteria (Table adapted from Kang et al. 2021 [12])

		1. Clinically excellent / very good	2. Clinically good (after polishing probably very good)	3. Clinically sufficient/Satisfactory (minor shortcomings, no unacceptable effects but not adjustable w/o damage to the tooth)	4. Clinically unsatisfactory (but repairable)	5. Clinically poor (replacement necessary)
Esthetic Properties	**Surface Luster**	1. Luster comparable to enamel	2.1 Slightly dull, not noticeable from speaking distance. 2.2 Some isolated pores.	3.1 Dull surface but acceptable if covered with film of saliva. 3.2 Multiple pores on more than one third of surface	4.1 Rough surface, cannot be masked by saliva film, simple polishing is not sufficient. Further intervention necessary. 4.2 Voids.	5.Very rough, unacceptable plaque retentive surface.
Esthetic Properties	**Surface Staining**	1. No surface staining	2. Minor surface staining, easily removable by polishing	3. Moderate surface staining that may also present on other teeth, not esthetically unacceptable	4. Unacceptable surface staining on the restoration and major intervention necessary for improvement	5. Severe surface staining and/or subsurface staining, generalized or localized, not accessible for intervention
Esthetic Properties	**Marginal Staining**	1. No marginal staining	2. Minor marginal staining, easily removable by polishing	3. Moderate marginal staining, not esthetically unacceptable	4. Pronounced marginal staining; major intervention necessary for improvement	5. Deep marginal staining, not accessible for intervention
Esthetic Properties	**Color Match and Translucency**	1. Good color match, no difference in shade and/or translucency	2. Minor deviations in shade and/or translucency	3. Distinct deviation but acceptable. Does not affect esthetics: 3.1 more opaque 3.2 more translucent 3.3 darker 3.4 brighter	4. Localized clinically deviation that can be corrected by repair: 4.1 too opaque 4.2 too translucent 4.3 too dark 4.4 too bright	5. Unacceptable. Replacement necessary
Esthetic Properties	**Esthetic anatomical form**	1. Form is ideal	2. Form is only slightly deviated from the normal	3. Form deviates from the normal but is esthetically acceptable	4. Form is affected and unacceptable esthetically. Intervention/correctio n is necessary	5. Form is unsatisfactory and/or lost. Repair not feasible/reasonabl e. Replacement needed

**Functional Properties**	**Fracture of material and retention**	1. No fractures /cracks	2. Small hairline crack	3. Two or more or larger hairline cracks and/or material chip fracture not affecting the marginal integrity or proximal contact	4. Material chip fractures which damage marginal quality or approximal contacts<br>4.2 Bulk fractures with partial loss (less than half of the restoration)	5. (Partial or complete) loss of restoration or multiple fractures
	**Marginal adaptation**	1. Harmonious outline, no gaps, no white or discolored lines	2.1 Marginal gap (<150 µm), white lines<br>2.2 Small marginal fracture removable by polishing<br>2.3 Slight ditching, slight step/flashes, minor irregularities	3.1 Gap <250µm not removable<br>3.2. Several small marginal fractures<br>3.3 Major irregularities, ditching or flash,steps	.1 Gap >250µm or dentine/base exposed<br>4.2. Severe ditching or marginal fractures<br>4.3 Larger irregularities or steps (repair necessary)	5.1 Restoration (complete or partial) is loose but in situ<br>5.2 Generalized major gaps or irregularities.
	**Radiographic examination (when applicable)**	1. No pathology, Harmonious transition between restoration and tooth	2.1 Acceptable material excess present.<br>2.2 Positive/negative step present at margin <150 µm	3. 1 Marginal gap < 250 µm.<br>3. 2 Negative steps visible < 250 µm. No adverse effects noticed.<br>3.3 Poor radiopacity of filling material.	4.1 Marginal gap >250 µm.<br>4.2 Material excess accessible but not removable.<br>4.3 Negative steps >250µm and reparable	5.1 Secondary caries, large gaps, large overhangs<br>5.2 Apical pathology<br>5.3 Fracture/loss of restoration or tooth.
	**Subject's View**	1. Entirely satisfied with esthetics and function.	2. Satisfied.<br>2.1 Esthetics<br>2.2 Function, e.g., minor roughness	3. Minor criticism but no adverse clinical affects.<br>3.1 Esthetic shortcomings.<br>3.2 Some lack of chewing comfort.<br>3.3 Unpleasant treatment procedure	4. Desire for improvement.<br>4.1. Esthetics<br>4.2 Function, e.g., tongue irritation. Reshaping of anatomic form or refurbishing is possible	5. Completely dissatisfied and/or adverse effects, including pain
**Biological Properties**	**Postoperative (hypersensitivity and tooth vitality**	1. No hypersensitivity, normal vitality.	2. Minor hypersensitivity for a limited period of time, normal vitality	3.1 Moderate hypersensitivity.<br>3.2 Delayed/mild sensitivity; no subjective complaints, no treatment needed	4.1 Intense hypersensitivity.<br>4.2 Delayed with minor subjective symptoms.<br>4.3 No clinical detectable sensitivity. Intervention necessary but not replacement	5. Intense, acute pulpitis or non vital tooth. Endodontic treatment is necessary and restoration has to be replaced

**Recurrence of caries (CAR), erosion, abfraction**	1. No secondary or primary caries	2. Small and localized<br>2.1. Demineralization<br>2.2. Erosion or<br>2.3. Abfraction	3. Larger areas of<br>3.1.Demineralization<br>3.2. Erosion or<br>3.3. Abrasion/Abfraction,no dentin exposure. Only preventive measures necessary	4.1 Caries with cavitation and suspected undermining caries<br>4.2 Erosion in dentine<br>4.3 Abrasion/abfraction in dentine. Localized and accessible can be repaired.	5. Deep caries or exposed dentin that is not accessible for repair of restoration.
**Tooth integrity (enamel cracks, tooth fractures)**	1. Complete integrity	2.1 Small Marginal enamel fracture (<150μm).<br>2.2 Hairline crack in enamel (<150 μm).	3.1 Marginal enamel defect <250μm<br>3.2 Crack <250μm;<br>3.3 Enamel chipping	4.1 Major marginal enamel defects; gap >250 μm or dentine or base exposed<br>4.2 Large cracks >250 μm, probe penetrates.<br>4.3. Large enamel chipping or wall fracture	5. Cusp or tooth fracture
**Adjacent mucosa**	1. Healthy mucosa adjacent to restoration	2. Healthy after minor removal of Mechanical irritations (plaque, calculus, sharp edges etc.	3. Alteration of mucosa but no suspicion of causal relationship with restorative material	4. Suspected mild allergic, lichenoid or toxic reaction.	5. Suspected severe allergic, lichenoid or toxic reaction.
